# An unusual thrombus in a patient with arrhythmogenic cardiomyopathy

**DOI:** 10.34172/jcvtr.025.33140

**Published:** 2025-03-18

**Authors:** Sezgin Atmaca, Arda Guler, İrem Türkmen, Sinem Aydın, Songul Ustundag, Gamze Babur Guler

**Affiliations:** ^1^Istanbul Mehmet Akif Ersoy Thoracic and Cardiovascular Surgery Training and Research Hospital, Department of Cardiology, Istanbul, Turkey; ^2^Istanbul Mehmet Akif Ersoy Thoracic and Cardiovascular Surgery Training and Research Hospital, Department of Radiology, Istanbul, Turkey; ^3^Medicine Hospital, Department of Cardiology, Istanbul, Turkey

**Keywords:** Anticoagulants, Arrhythmogenic cardiomyopathy, Cardiomyopathy, Echocardiography, Genetics, Cardiovascular diseases, Thrombus

## Abstract

Herein we present a case of a right ventricular (RV) thrombus in a patient with arrhythmogenic cardiomyopathy (ACM). The 24-year old female patient was diagnosed with ACM after echocardiography, genetic test and cardiac magnetic resonance imaging. Interestingly, at echocardiography, an unusal thrombus formation was detected at RV lateral wall. Also, CMR confirmed the thrombus and oral anticoagulant therapy was started. During the patient’s follow-ups, it was observed that the imaging consistent with the reported thrombus disappeared after effective anticoagulant treatment. After the diagnosis was confirmed with genetic tests, an implantable cardioverter-defibrillator (ICD) was implanted in the patient with a high sudden cardiac death (SCD) risk score. Even in arrhythmogenic right ventricular cardiomyopathy patients thrombi are rarely reported. However, the development of imaging techniques may enable more frequent detection and effective treatment of thrombi in these patients.

## Introduction

 Left ventricular (LV) thrombi are frequently encountered in our clinical practice and usually associated with the underlying myocardial disease. However, right ventricular (RV) thrombi are rare and more difficult to diagnose.^1^ Imaging of the right ventricle in transthoracic echocardiography (TTE) can be difficult and the importance given to right ventricular pathologies is lower than that of the left ventricle. Although RV thrombi can be observed in the presence of severe RV dilatation, wall motion defect and cardiomyopathy, the cases reported in the literature are very limited.^2-4^ Arrhythmogenic cardiomyopathy (ACM) is a rare disease of genetic origin that can present with life-threatening arrhythmias with RV dilatation and wall motion abnormalities.^5^ Due to the presence of RV dilatation, aneurysms, and wall motion abnormalities, we may rarely detect RV thrombi in the course of ACM. Hence, in this case, we will try to present a patient with ACM with atypically localized RV thrombus formation.

## Case report

**Figure 1:**
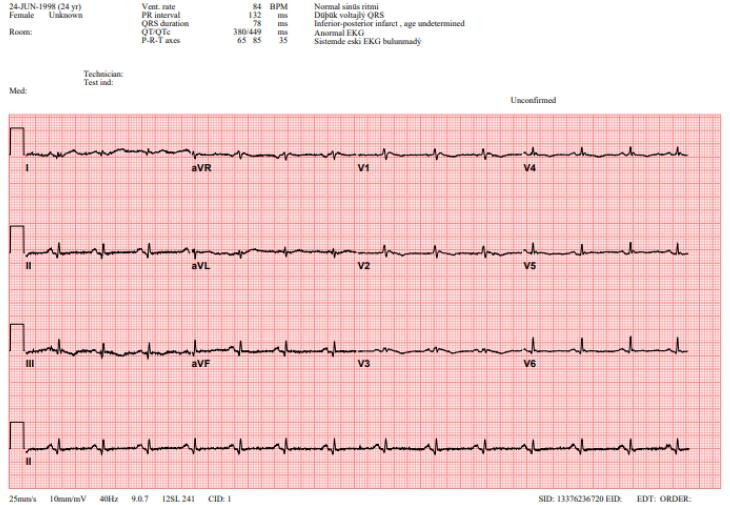
ECG Showing Sinus Rhythm and T Wave Negativity. The patient’s ECG reveals sinus rhythm with T wave negativity observed in leads V1 to V5, which may indicate regional myocardial changes. FIGUR LEGEND: ECG: Electrocardiogram.

**Figure 2:**
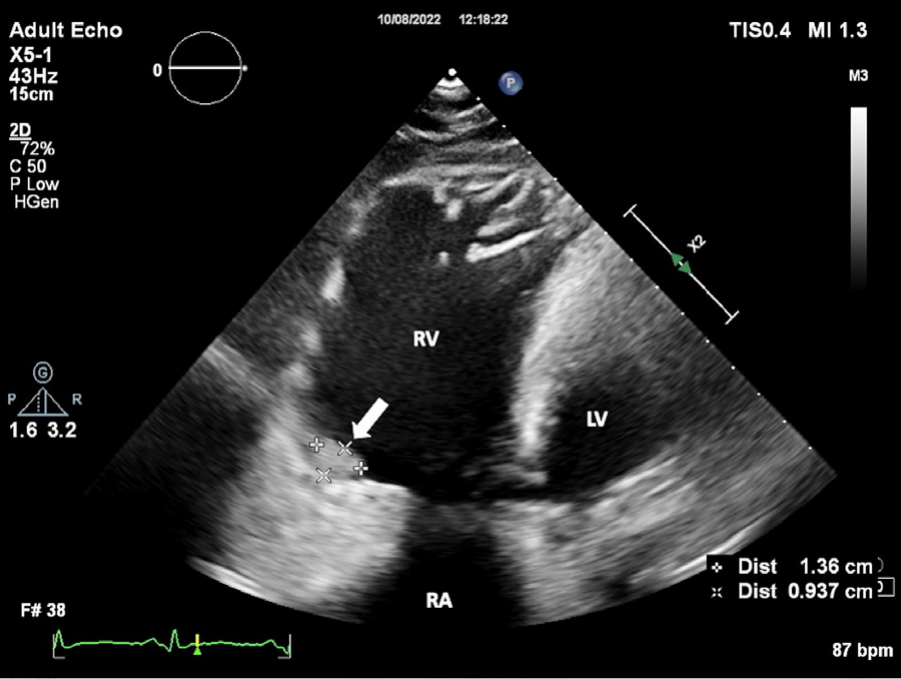
Wall Irregularity of the Right Ventricle. Transthoracic echocardiography (TTE) shows the wall irregularity of the right ventricle. FIGUR LEGEND: TTE: Transthoracic Echocardiography, RA: Right Atrium, RV: Right Ventricle, LV: Left Ventricle.

**Figure 3:**
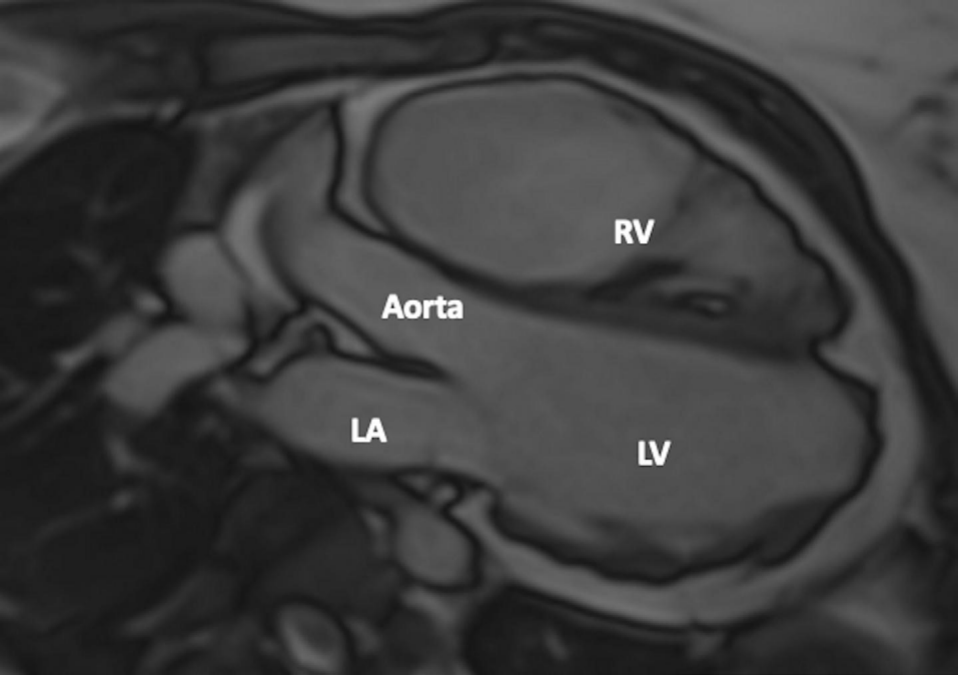
Wall Irregularity in Both Right and Left Ventricles. CMR 3-chamber view shows wall irregularity in both the right and left ventricles. FIGUR LEGEND: CMR: Cardiac Magnetic Resonance, LA: Left Atrium, RV: Right Ventricle, LV: Left Ventricle.

**Figure 4:**
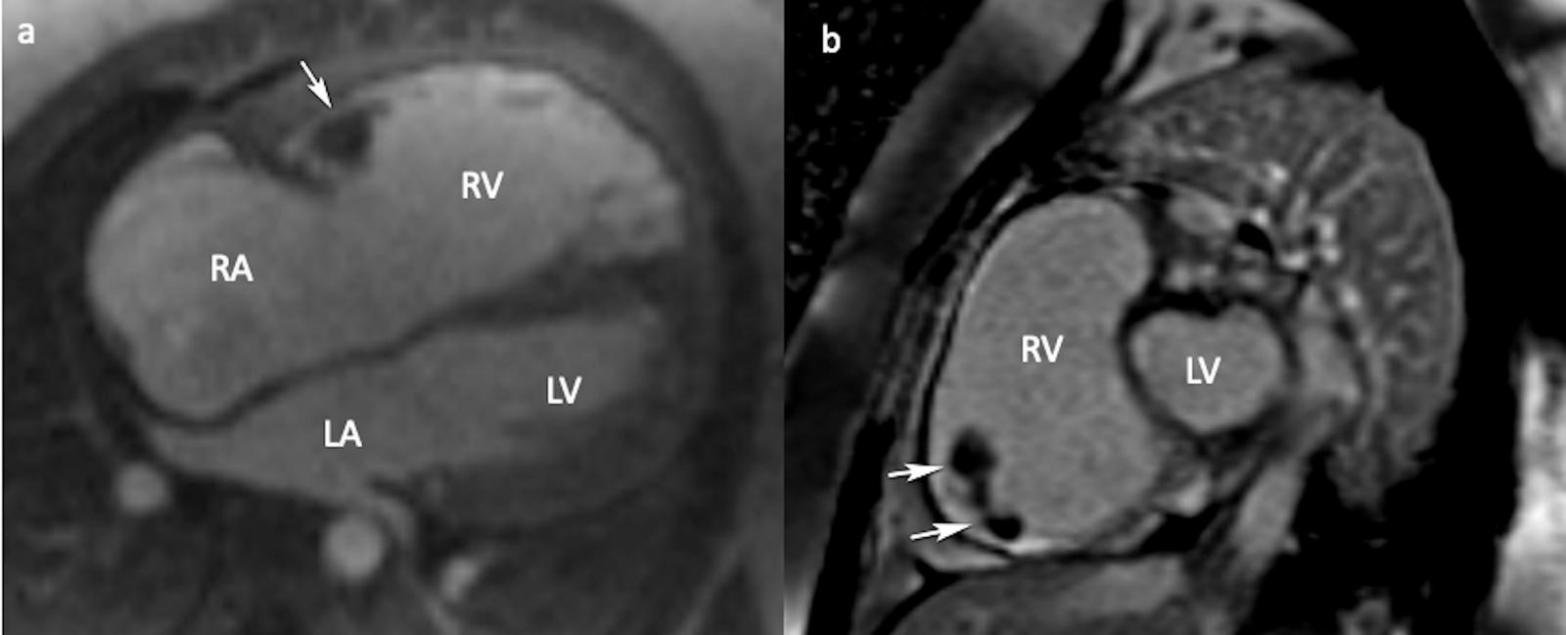
CMR 4-chamber (a) and short-axis (b) LGE views showing non contrast enhancing thrombus (arrows). (CMR: Cardiac magnetic resonance, LGE: Late gadolinium enhancement, RA: Right atrium, LA: Left atrium, RV: Right ventricle, LV: Left ventricle)

 A 24-year-old female patient was admitted to our tertiary center with chest pain. She had no family history of cardiac disease or sudden cardiac death. Her electrocardiogram (ECG) was sinus rhythm and T wave negativity was detected in leads V1-5. (Figure 1) TTE showed a slight decrease in LV ejection fraction as 45%, prominently dilated right heart chambers, decreased RV functions (TAPSE: 11, tricuspid annular peak systolic velocity: 7.7), severe tricuspid regurgitation (Supplementary file 1, Video 1). Additionally, an image of 13.6 x 9.3 mm, hyperechoic, consistent with thrombus was observed in the lateral wall of the RV (Figure 2). No thromboembolism was reported in the pulmonary CTA. The patient underwent cardiac magnetic resonance imaging (CMR) for morphological and functional assessment of the heart. The CMR images showed enlarged RV and right atrium, severe hypokinesia of the RV, dyskinesia in the free wall of RV and paradoxical movement in interventricular septum, and wall irregularity in both ventricles (Figure 3). In the cardiac MRI, The LV ejection fraction was 49%, the LV end-systolic volume was 143 mL, end-diastolic volume was 73 mL, and stroke output was 60 mL. The LV end-diastolic volume index (LVEDVI) was 74 mL/m^2^, the LV end-systolic volume index (LVESVI) was 42 mL/m^2^. The RV ejection fraction was 20%, the RV end-systolic volume was 430 mL, end-diastolic volume was 343 mL, and stroke output was 87 mL. The RV end-diastolic volume index (RVEDVI) was 221 mL/m^2^, the RV end-systolic volume index (RVESVI) was 176 mL/m^2^. The late gadolinium images depicted diffuse enhancement in the right ventricular free wall and inferior wall. The left ventricular inferior septum showed midwall enhancement. A nodular mass was noted on the lateral wall of RV in the vicinity of the tricuspid valve which did not uptake gadolinium and was compatible with thrombus (Figure 4). As a result of these findings, genetic analysis was performed with a high suspicion of arrhythmogenic cardiomyopathy. The genetic analysis revealed a homozygous nonsense mutation in the desmoglein-2 gene, proven to be pathogenic in databases, and the diagnosis of arrhythmogenic cardiomyopathy was definitively confirmed with the findings from TTE, ECG, CMR, and genetic tests, which are part of the aforementioned Padua criteria.

 Concerning the RV thrombus, we initiated warfarin therapy and monitored the INR levels closely to ensure its efficacy. After achieving the targeted INR, a resolution of the thrombus-like mass was observed in the follow-up echocardiography after 3 months (Supplementary file 2, Video 2). ICD is contraindicated in situations where there are reversible causes (like myocardial ischemia, sepsis, hypoxia, electrolyte imbalance, electrocution, etc.) leading to VT/VF. For this reason, an ICD implantation was performed in our patient after the thrombus-like mass was resolved following medical treatment. An implantable cardioverter defibrillator (ICD) was implanted to the patient for primary prevention. The patient remains asymptomatic during follow-up visits and diligently attends scheduled appointments. Furthermore, proactive measures have been taken by inviting the patient’s first-degree relatives for cardiac disease screening.

## Discussion

 RV thrombus formation is rare, even in ACM with dominantly RV involvement. Wlodarska et al reported an annual incidence of 0.5 thromboembolic events per 100 patients in 126 confirmed Arrhythmogenic right ventricular cardiomyopathy (ARVC) patients at a mean follow-up of 99 months.^6^ In ARVC patients, slowed blood flow with dilated and hypokinetic RV are important risk factors for thromboembolism. Also, a study by Wu et al showed that female gender and low LVEF were also associated with high thrombus prevalence.^7^

 In previously reported cases, most of the RV thrombi were located in the RV apex (2,8). In our case, interestingly, we detected the thrombus on the lateral wall. Since RV evaluation with TTE may be insufficient, evaluation of ACM, especially with RV involvement, patients with CMR is of great importance. CMR is an important diagnostic tool in showing wall motion abnormalities, and thanks to its high resolution, it enables the detection of structures such as thrombus that may be missed in TTE.

 Treatment strategy in the presence of RV thrombus has been defined for pulmonary embolism, but there are no guidelines for treatment in the presence of cardiomyopathy.^8^ It was showed that RV thrombi were regressed with anticoagulation and the importance of early and adequate anticoagulation was emphasized in a study by Akdis et al^9^ Surgical thrombectomy may rarely be considered in selected cases that do not resolve despite anticoagulant therapy. Likewise, although the optimal duration for anticoagulation has not been determined, life-long therapy seems to be a reasonable option. There is no study on which anticoagulant agent to choose. Although it is claimed that NOACs can be effective in this regard, we preferred VKA with the classical approach and determined the follow-up under the control of INR as a more accurate approach.

## Conclusion

 In summary, Arrhythmogenic Cardiomyopathy (ACM) is a rare cardiac disease associated with sudden cardiac death, and its diagnosis involves multimodality imaging and genetic analysis. In ACM patients right ventricular thrombi can also rarely be seen. Therefore, it is of great importance that the echocardiographic evaluation is carried out in detail and meticulously, and, if available, to add CMR for diagnostic workup. In patients with intracardiac thrombus, early and effective use of oral anticoagulants and close follow-up are crucial in terms of prognosis and prevention of thromboembolism.

## Competing Interests

 The authors have nothing to declare.

## Ethical Approval

 Written informedconsent was obtained from the patient for the publication of thiscase report and accompanying images.

## Supplementary Files


Video 1: The video shows biventricular dysfunction, marked right heart dilatation, severe tricuspid regurgitation, and a thrombus in the lateral wall of the right ventricle as demonstrated by TTE. (TTE: transthoracic echocardiography)

Video 2: The video shows the regression of the thrombus-like mass on follow-up echocardiography 3 months after achieving the targeted INR.
